# Kinetics of Thymocyte Subset Development and Selection Revealed by Cyclosporin A Treatment

**DOI:** 10.1155/1995/61309

**Published:** 1995

**Authors:** Russell D.J. Huby, Ray Hicks, Lindsey K. Goff

**Affiliations:** 1 Imperial Cancer Research Fund Human Tumour Immunology Group The Courtauld Institute for Biochemistry London W1P, 8BT United Kingdom; 2 Department of Cellular Immunology National Institute for Medical Research The Ridgeway Mill Hill London United Kingdom

**Keywords:** Cyclosporine A, CsA, kinetics, apoptosis, thymocyte, development, autoreactivity, CD45

## Abstract

Cyclosporin A (CsA) inhibits the development of mature thymocytes from their CD4^+^ CD8^+^ precursors, but may allow autoreactive cells to mature. Using 3-color flow cytometry, we have followed the progressive development of thymocytes, including potentially autoreactive cells, during CsA treatment. Numbers of CD4^+^ CD8^+^ CD3^high^ thymocytes dropped immediately, suggesting that the generation of these mature thymocyte precursors, normally dependent upon positive selection, was inhibited by CsA. Numbers of CD4^-^ CD8^+^ thymocytes also declined rapidly, but CD4^-^ CD8^+^ thymocytes were unaffected lfor 2 days, suggesting that the mature single-positive subsets are not symmetrically derived from a common GsA-sensitive precursor. An exceptional subset of CD8 SP thymocytes, expressing CD45RA, did not respond to CsA for about 10 days, indicating that they are distantly derived from a CsA-sensitive precursor. Apoptosis of TCR-V*β*3^+^ thymocytes caused by *Mtυ*-6, quantified according to the down-regulation of CD4 and CD8 on immature thymocytes, was partially inhibited by CsA, to maximal effect within 24 hours. This did not, however, facilitate their development into mature thymocytes.


*Developmental Immunology*, 1995, Vol 4, pp. 117–126
Reprints available directly from the publisher
Photocopying permitted by license only

© 1995 Harwood Academic Publishers GmbH
Printed in Singapore

### Kinetics of Thymocyte Subset Development and Selection Revealed by Cyclosporin A Treatment

RUSSELL D.J. HUBY,* RAY HICKS and LINDSEY K. GOFF

*Imperial Cancer Research Fund, Human Tumour Immunology Group, The Courtauld Institute for Biochemistry, London W1P, 8BT, UK*

Cyclosporin A (CsA) inhibits the development of mature thymocytes from their $CD4^+CD8^+$ precursors, but may allow autoreactive cells to mature. Using 3-color flow cytometry, we have followed the progressive development of thymocytes, including potentially autoreactive cells, during CsA treatment. Numbers of $CD4^+CD8^+CD3^{high}$ thymocytes dropped immediately, suggesting that the generation of these mature thymocyte precursors, normally dependent upon positive selection, was inhibited by CsA. Numbers of $CD4^+CD8^-$ thymocytes also declined rapidly, but $CD4^-CD8^+$ thymocytes were unaffected lfor 2 days, suggesting that the mature single-positive subsets are not symmetrically derived from a common GsA-sensitive precursor. An exceptional subset of CD8 SP thymocytes, expressing CD45RA, did not respond to CsA for about 10 days, indicating that they are distantly derived from a CsA-sensitive precursor. Apoptosis of TCR-$V\beta3^+$ thymocytes caused by *Mtv-6*, quantified according to the down-regulation of CD4 and CD8 on immature thymocytes, was partially inhibited by CsA, to maximal effect within 24 hours. This did not, however, facilitate their development into mature thymocytes.

KEYWORDS: Cyclosporine A, CsA, kinetics, apoptosis, thymocyte, development, autoreactivity, CD45.

### INTRODUCTION

Cyclosporin A (CsA) is an immunosuppressive drug known to influence the development of thymocytes. At a molecular level, CsA target sites have been identified as cyclophilin and calcineurin (Fischer et al., 1989; Takahashi et al., 1989; Liu et al., 1991; Clipstone and Crabtree, 1992; O'Keefe et al., 1992). Their significance at stages of T-cell development, however, remains to be determined. Nevertheless, treatment with CsA presents a useful tool with which to dissect normal thymocyte development. Studying the effect on thymocytes at a single time point after long-term GsA treatment has identified subsets that are downstream of CsA-sensitive populations and the extent to which they are sensitive. Most conspicuously, the development of mature SP thymocytes is greatly inhibited (Gao et al., 1988; Jenkins et al., 1989) leading to atrophy of the medulla (Kai and Franklin, 1983; Kanariou et al., 1989).

It is reported that thymocytes bearing T-cell receptor $V\beta$-segments (TCRs) reactive with self-superantigens become detectable amongst mature thymocytes (Gao et al., 1988; Jenkins et al., 1989), and in the periphery (Urdahl et al., 1992), where they could potentially mediate an autoimmune response. Such cells (Abe et al., 1988; MacDonald et al., 1988; Pullen et al., 1988) are normally eliminated by clonal deletion during thymic development (Kappler et al., 1987; Fry et al., 1989; Hodes et al., 1989; Schwartz, 1989), preventing their maturation and entry into the medulla (Hengartner et al., 1988; Sha et al., 1988; Hugo et al., 1991a). Clonal deletion is effected via apoptosis (Jenkinson et al., 1989), which is typified by morphological changes, and fragmentation of genomic DNA into multimers of ~180 base-pair units (Wyllie et al., 1984). Immature thymocytes undergoing apoptosis additionally down regulate expression of CD4 and CD8, but retain an intact cell membrane, thereby forming a population that can be identified by flow cytometry

*Corresponding author. Present address: Department of Cellular Immunology, National Institute for Medical Research, The Ridgeway, Mill Hill, London, UK.

(Swat et al., 1991a, 1991b) and followed using TCR-V$\beta$-specific antibodies (Huby and Goff, manuscript in preparation).

Experimentally, a condition similar to graft-versus-host disease can be initiated in young rats (Glazier et al., 1983; Hess et al., 1985) or mice (Cheney and Sprent, 1983; Bryson et al., 1989; Sakaguchi and Sakaguchi, 1989) by treating them with CsA after lethal irradiation and bone marrow reconstitution, and then withdrawing the drug. The disease can be initiated in secondary irradiated hosts by the transfer of peripheral T cells or thymocytes from these animals (Sorokin et al., 1986; Beschorner et al., 1988a), or from the thymus of nonirradiated but CsA-treated donors (Sakaguchi and Sakaguchi, 1988). This suggests that CsA induces the generation of autoreactive thymocytes that are exported to the periphery while the drug is still being administered (Wodzig et al., 1991) and can initiate an autoimmune reaction when it is withdrawn. The cells responsible for such CsA-induced autoimmunity are likely to be polyclonal, and directed toward true self-antigens conventionally presented by MHC. In contrast, superantigen-reactive cells express specific TCR-V$\beta$ segments that recognize superantigen bound to MHC class II outside the antigen-binding groove (Dellabona et al., 1990; Choi et al., 1992). The latter are attractively simple to study, although their behavior may not necessarily reflect that of conventional antigen-responsive cells.

It has been speculated that the inhibition of mature thymocyte development occurs during the $CD4^+CD8^+$ (double-positive) stage; however, by observing subsets at a single time point after CsA treatment, it is not possible to confirm this. We have studied the *kinetics* of thymocyte subsets in the presence of CsA, using three-color flow cytometry as a means of pinpointing multiple stages of thymocyte development. In addition, three-color flow cytometry in this study has enabled the fate of TCR-V$\beta 3^+$ thymocytes to be followed, these cells normally being subject to deletion by *Mtv-6* in BALB/c mice (Abe et al., 1988; MacDonald et al., 1988; Pullen, et al., 1988).

A kinetic study not only allows CsA-sensitive subsets to be identified, but also reveals the time taken for a subset to develop from its CsA-sensitive precursor (Fig. 1, point a). Thus, the faster a subset is derived from a CsA-sensitive precursor, the more rapidly it will be lost during treatment. In addition, the initial rate at which a subset is lost, once its immediate precursor has been blocked, will be equal to the rate of flux into that subset (Fig. 1, point b). Heterogeneity of the subset will be reflected by a nonlinear loss of cells with time (Fig. 1, point c) and the size of the subset remaining will be a measure of the efficacy of the CsA treatment (Fig. 1, point d).

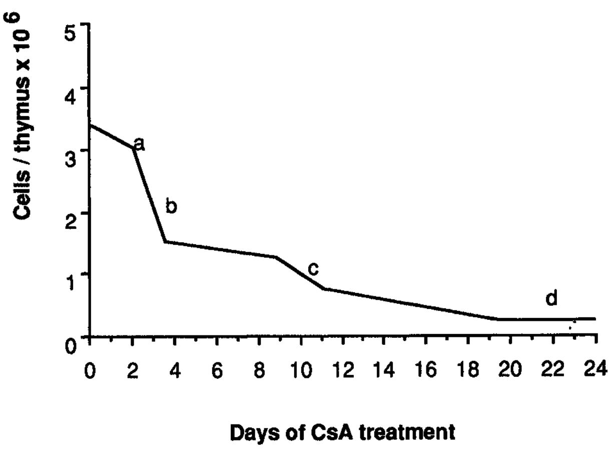

FIGURE 1. Representation of a kinetic study on the effect of CsA on a thymocyte subset. The time taken to reach point a, the point of decline in cell number, indicates the temporal proximity of the subset to its CsA-sensitive precursor. The gradient of point b, the initial loss of cells from the subset, indicates the rate of flux into that subset. Fluctuation in the size of the subset, point c, indicates heterogeneity within that subset. The size of the subset remaining, point d, indicates the limit of depletion of the subset and is a measure of the efficacy of the CsA treatment.

The interpretation of these results requires that the effects of CsA on specific thymocyte subsets are direct. Although CsA can affect the thymic microenvironment, histological studies have shown such changes to occur relatively slowly, and therefore unlikely to account for CsA's effect on thymocytes. A kinetic study is again advantageous, demonstrating that different thymocyte subsets respond to CsA over very different times spans, an unlikely result if CsA were acting though a common toxic effect, or through effects on the thymic stroma.

Here we show that the development of the most mature $CD4^+CD8^+CD3^{high}$ cortical thymocytes is inhibited by CsA and that CD4 and CD8 SP cells are not symmetrically derived from CsA-sensitive DP precursors. Also, among mature CD8 SPs, two subpopulations with distinct sensitivity to CsA are revealed and they are distinguishable by expression of CD45RA. The apoptosis of TCR-V$\beta 3^+$ thymocytes is partially inhibited by CsA, but this does not permit TCR-V$\beta 3^+$ to mature.

### MATERIALS AND METHODS

### Mice

Female BALB/c (*Mtv-6*$^+$) and B10.D2 (*Mtv-6*$^-$) mice were obtained from ICRF, and used when 6–8 weeks old. CsA (Sandimmun; Sandoz UK Ltd., Leeds) at 50 mg/ml was dissolved at 1 mg/ml in 0.9% saline, and given I.P. at 10 mg/kg/day as appropriate. Controls received saline only. Three animals were used per time point.

### Antibodies

Conjugated antibodies used were anti-CD4-PE and CD8-FITC (Becton-Dickinson, Oxford, UK); anti-CD3 (ε-chain)-biotin and anti-TCR-Vβ3-biotin (both from PharMingen, San Diego); anti-CD45RA antibody RA3-2C2 (ATCC) was purified from cell culture supernatant using an anti-rat Ig-Sepharose column and biotinylated using standard procedures.

### Staining and Flow Cytometry

Thymocytes were obtained by teasing freshly excised organs in 5 ml of cold phosphate-buffered saline (PBS) containing 1% bovine serum albumin (BSA) and 0.1% sodium azide. Total thymocyte counts were estimated using a haemocytometer, using trypan blue to exclude dead cells. Three-color staining of $1 \times 10^6$ cells to reveal CD4, CD8 and CD3, CD45RA, or TCRVβ3 was carried out by incubation with a cocktail of the appropriate conjugated antibodies, followed by Streptavidin-Cychrome (PharMingen). Cells were washed twice in suspension buffer between incubations, which were of 30–60 min. each, carried out on ice. After staining, cells were washed and then fixed in 1% formaldehyde in PBS. Single- and double-color staining controls were carried out for the purposes of color compensation as appropriate. A FACScan cytometer (Becton-Dickinson) with FACScan software was used to analyze 10,000 cells per test. Debris and erythrocytes were eliminated during acquisition by using a generous forward/side scatter dot-plot gate that included apoptotic cells.

### Flow Cytometric Analysis by Cross-Correlation

Four possible subsets of CD4$^-$8$^+$ thymocytes can be defined according to their coexpression of CD3 and CD45RA; CD3CD45RA$^-$, CD3CD45RA$^+$, CD3CD45RA$^-$, and CD3CD45RA$^+$. Of these, only CD3$^-$45RA$^+$ thymocytes do not occur in measurable numbers *in vivo* (Goff and Huby, 1992). The size of the other subsets was determined by cross-correlation as follows: Total CD4$^-$8$^+$ was determined from double staining for CD4 and CD8. By using triple staining and gating on CD4$^-$8$^+$ cells, the fraction bearing CD45RA and CD3, respectively, was determined directly for appropriately stained samples. Cells not accounted for by either the CD45RA$^+$ subset or the CD3$^-$ subset were taken to be CD3$^+$CD45RA$^-$.

### Analysis of Flow Cytometric Dot Plots

From samples triple-stained for CD4, CD8, and TCR-Vβ3, 1,000 TCR-Vβ3$^+$ events were displayed on dot plots of CD4 vs TCR and CD8 vs TCR. Images were captured as black-and-white drawings at 100 dots per inch using a Hewlett-Packard Scanjet IIc scanner, and analyzed using Deskpaint 3.20 and Image 1.47 software run on a Macintosh IIsi. Captured images were blurred by convoluting with 15 x 15 gaussian smoothing and a preset intensity threshold applied to distinguish a discrete subset of apoptotic cells. Calculation revealed the angle to horizontal of the best-fit oval encompassing this subset.

### RESULTS

### Stages of Thymocyte Maturation Inhibited by CsA

Treatment of BALB/c mice with CsA at 10 mg/kg/day for 23 days was found to be effective in ablating the majority of CD4 SP and about half of the CD8 SP thymocytes (Fig. 2). Further subsetting was possible by detecting CD3 as a third color parameter. Numbers of CD4$^-$CD8$^-$CD3$^-$ (triple-negative) and CD4$^-$CD8$^-$ intermediates were moderately and slowly reduced during treatment, suggesting that intrathymic precursors with limited capacity for self-renewal were partially depleted over the period of the experiment (Fig. 3a). Nevertheless, numbers of their CD4$^+$CD8$^+$ progeny fell by ~50% within 4 days to a level that was subsequently maintained (Fig. 3b). Within the DP subset, numbers of CD3$^{high}$ thymocytes fell rapidly within 2 days of commencing treatment and thus represented the most CsA-sensitive subset within the thymus, indicating that

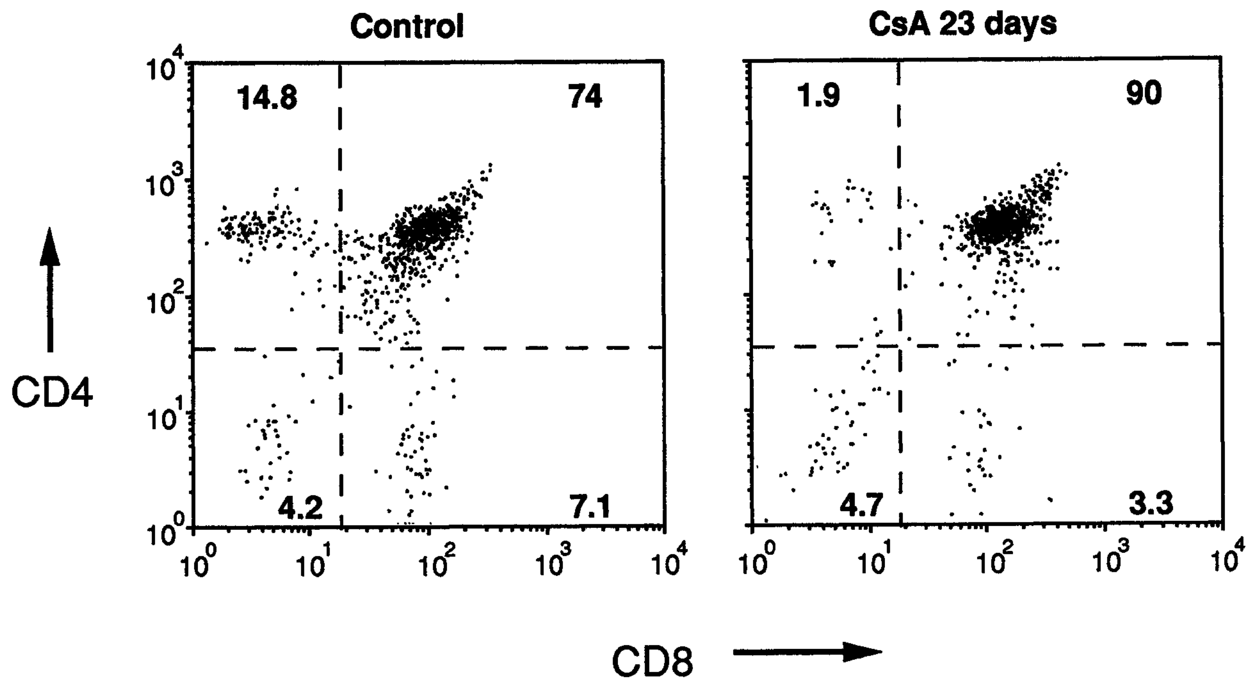

FIGURE 2. The effect of long-term CsA treatment on thymocyte subsets defined by CD4 and CD8 expression. Numbers show percentage of cells in subsets defined by the quadrants shown for a representative individual. 1,000 events are displayed.

development from their immediate precursors was inhibited (Fig. 3b).

CsA had an immediate effect on the number of CD4 SP thymocytes, with numbers dropping exponentially, consistent with CsA inhibiting the generation of their immediate $CD4^+CD8^+CD3^{high}$ precursors (Fig. 3c). Numbers of CD8 SP thymocytes, however, do not drop over the first 2 days (Fig. 3c). This implies that differentiation of their immediate precursors (also $CD4^+CD8^+CD3^{high}$ thymocytes) is insensitive to CsA, whereas that of an earlier precursor ~2 days "upstream" is sensitive. The loss of mature CD8 SP thymocytes was biphasic, owing to a heterogeneity that could be distinguished according to CD45RA expression; $CD45RA^-$ cells disappeared rapidly after an initial 2-day lag whereas $CD45RA^+$ cells were unaffected by CsA for the first 10 days of treatment, after which their numbers declined rapidly (Fig. 3d).

By gating on $CD3^{high}$ thymocytes, cells in transit from the $CD4^+CD8^+$ to the CD4 and CD8 SP subsets can be demonstrated (Fig. 4). Consistent with this analysis, the CD4 SPs, but not CD8 SPs were discrete from the $CD4^+CD8^+$ subset after 2 days of CsA treatment (cf. Fig. 3c). The CD8 SP subset was discrete by day 7, before the numbers of $CD8^+CD45RA^+$ thymocytes had started to decline (Fig. 3d), indicating that the latter were not directly derived from this DP subset.

### CsA Inhibits the Apoptosis of Autoreactive Thymocytes

Immature thymocytes undergoing apoptosis down-regulate CD4 and CD8 expression (Swat et al., 1991a, 1991b), and this phenomenon can be observed amongst TCR-$V\beta3^+$ thymocytes *in vivo* as a response to *Mtv-6* (Huby and Goff, manuscript in preparation). This causes an increase in the proportion of $CD4^{dim}CD8^{dim}$ thymocytes (Fig. 5), but quantification of their relative numbers is arbitrary and insensitive. We have found that down-regulation of CD4 and CD8 is concomitant with up-regulation of T-cell receptor, producing a characteristic profile for TCR-$V\beta3^+$ cells in dot plots of $V\beta3$ vs CD8 and $V\beta3$ vs CD4 (Fig. 5). Here, this profile was quantified using an image analysis program, as described in Materials and Methods. A single dose of CsA at 10 mg/kg led to a maximal effect on the apoptotic phenotype of TCR-$V\beta3^+$ immature thymocytes within 24 hours of administration. Thus, the angle corresponding to down-

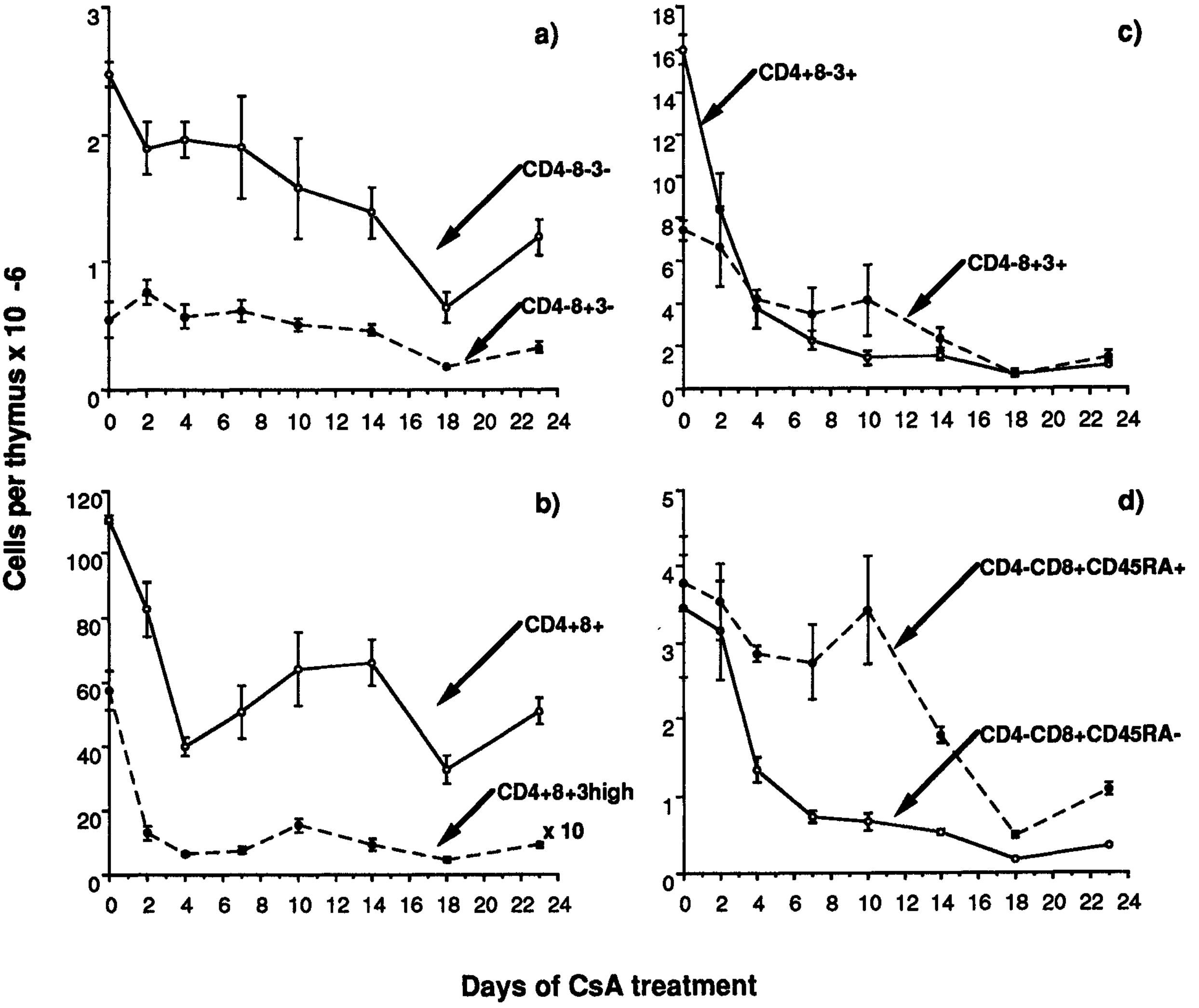

FIGURE 3. Progressive effect of CsA on the mean size of thymocyte subsets defined by triple staining for CD4, CD8, and CD3/CD45RA. 3 animals per time point. Note different vertical scales have been used, and note particularly that numbers for $CD4^+CD8^+CD3^{high}$ thymocytes have been multiplied by 10 to fit the same scale as total $CD4^+CD8^+$ thymocytes. Error bars = ±S.E.M.

regulation of CD8 among developing thymocytes up-regulating TCR-V$\beta3^+$ was reduced by 62% ($p = 0.012$), and that corresponding to down-regulation of CD4 by 30% ($p = 0.005$) (Fig. 5 and 6). Similar results were seen for as long as CsA was given (Fig. 6). Within 3 days of ceasing injections, however, the apoptotic phenotype of TCR-V$\beta3^+$ thymocytes was restored ($p = 0.14$ and $p = 0.53$ for CD4 and CD8 angles respectively, compared to untreated controls, Fig. 6). It should be noted that with CsA treatment, TCR-V$\beta3^+$ thymocytes did ultimately develop a $CD4^{dim}CD8^{dim}$ phenotype, albeit when they expressed higher levels of TCR, corresponding to a greater level of maturity; the overall fraction of cells with a $CD4^{dim}CD8^{dim}$ phenotype was therefore unaffected (Fig. 5).

### Mature Autoreactive Thymocytes Fail to Develop in CsA-Treated Mice

Using three-color staining, we determined the frequency of TCR-V$\beta3^+$ cells within subsets defined by CD4 and CD8, during CsA treatment. The fraction of thymocytes expressing TCR-V$\beta$3 was unaffected by CsA, the vast majority expressing low levels of this antigen, and belonging to the DP

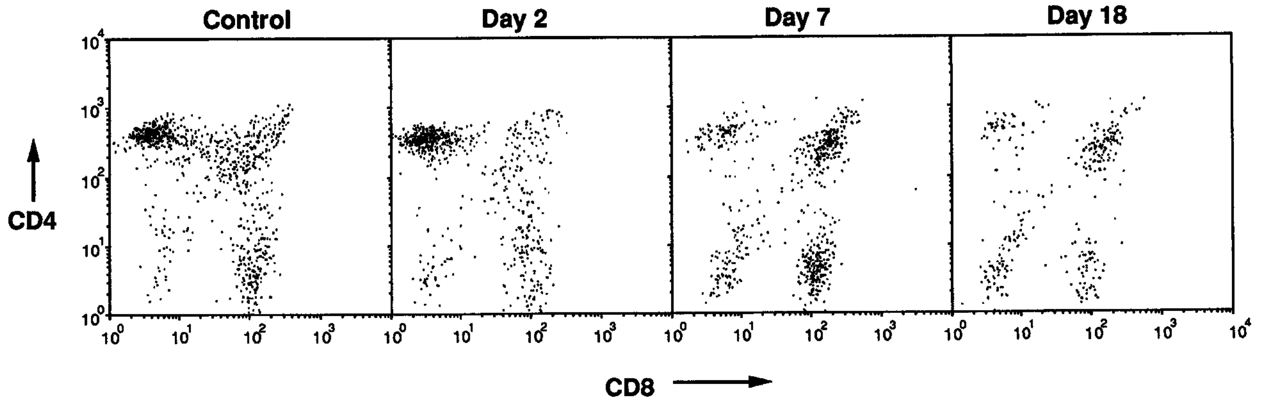

FIGURE 4. Do plots showing changes in the distribution of CD3$^{high}$ thymocytes within subsets defined by CD4 and CD8 after various periods of CsA treatment. The number of events displayed is proportional to the fraction of thymocytes expressing high levels of CD3; high is defined as the level expressed by CD3$^+$ SP thymocytes in control animals.

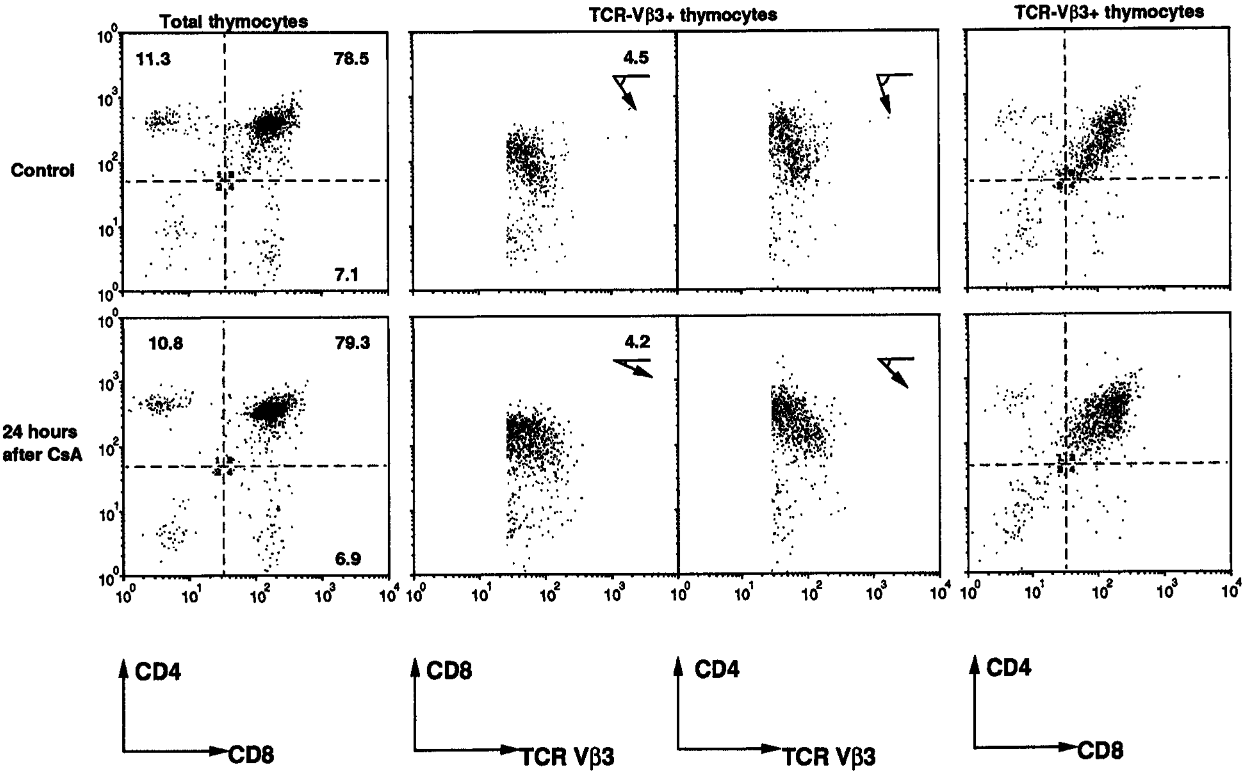

FIGURE 5. Effect of CsA on the down-regulation of CD4 and CD8 on TCR-V$\beta$3$^+$ thymocytes undergoing apoptosis induced by *Mtv-6*. Arrows show the approximate angle measured in each case in order to quantify the apoptotic phenotype. 1,000 events per plot are displayed. Numbers within dot plots represent the percentage of cells within the corresponding subpopulation.

subset (data not shown). Absolute numbers of TCR-V$\beta$3$^+$ thymocytes dropped for all subsets, particularly the mature SPs (Fig. 7). Thus, after 23 days of CsA, fewer than 6,000 mature TCR-V$\beta$3$^+$ SP cells per thymus were estimated to be present.

### DISCUSSION

Using three-color flow cytometry to analyze the kinetics of thymocytes treated *in vivo* with CsA, we have been able to define more specifically the thymocyte subsets that are affected by this drug and to determine the relationships between subsets and CsA-sensitive stages of development. We have found that numbers of early thymocytes, belonging to the CD4⁻CD8⁻CD3⁻ subset (Crispe et al., 1987; Scollay et al., 1988) and their immediate CD4⁻CD8⁻CD3⁻ progeny (Shortman et al., 1988) decline slowly in the presence of CsA. This is consistent with previous results showing that pre-prothymocytes are sensitive to CsA (Huby et al., 1989), and that intrathymic prothymocytes have a large but limited capacity for self-renewal (Frey et al., 1992). $CD4^-CD8^-CD3^+$ thymocytes, thought to constitute a side lineage with a slow turnover (Egerton et al., 1990) and a CsA-sensitive precursor (Takahama et al., 1991), declined with very similar kinetics to $CD4^-CD8^-CD3^-$ thymocytes (unpublished data).

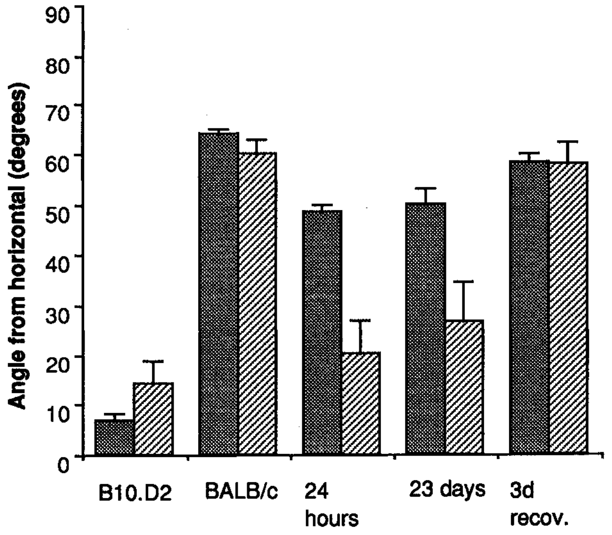

FIGURE 6. Inhibition of the apoptotic phenotype by CsA. Bars represent the mean angle of the TCR-Vβ3⁺ subset to horizontal, as demonstrated in Fig. 4, for 3 to 6 mice per group. Grey = vs. CD4; hatched = vs. CD8; error bars = ±S.E.M.; 3d recov. = thymocytes from mice treated with CsA for 18 days and then untreated for 3 days. To facilitate quantification of this subset in B10.D2 (*Mtv-6⁻*) control mice, SP thymocytes were first gated out.

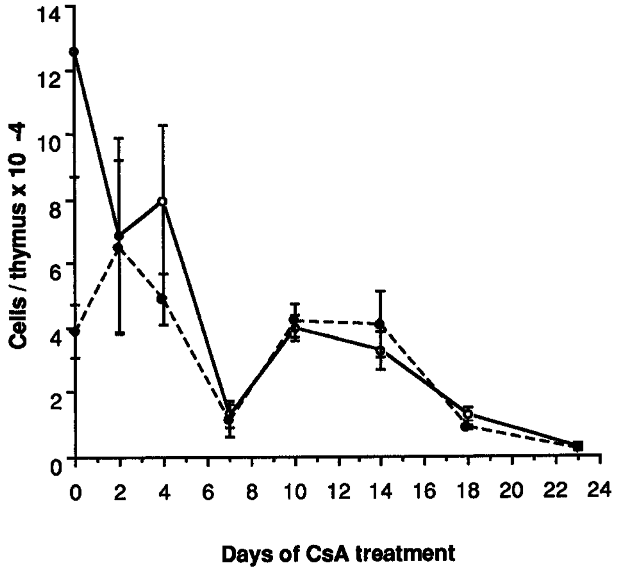

FIGURE 7. Progressive effect of CsA on mean numbers of TCR-Vβ3⁺ SP cells per thymus. Solid line = CD4 SP; dotted line = CD8 SP; error bars = + S.E.M.

In BALB/c mice, about 50% of the $CD4^+CD8^+$ subset was lost within 4 days of commencing CsA treatment, primarily accounting for a similar loss of thymic weight over the same period. Placebo-injected controls were essentially unaffected (data not shown). Within the $CD4^+CD8^+$ subset, the $CD3^{high}$ fraction underwent very rapid depletion, falling to minimal levels within 2 days. Such a rapid decline indicates that this subset is very transient, as described elsewhere (Hugo et al., 1991b), and that its immediate precursors are CsA-sensitive. It has been suggested elsewhere that the $CD4^+CD8^+CD3^{high}$ subset is the immediate progeny of $CD4^+CD8^+CD3^{low}$ cells (Penit, 1990; Huesmann et al., 1991). CsA must also partially block the development of DPs further upstream to account for a loss of over half these cells by day 4 after the start of treatment. CsA therefore may inhibit partially the development of $CD4^+CD8^+$ thymocytes from earlier precursors, or it may affect the kinetics of cell development within that subset.

Because most $CD4^+CD8^+CD3^{high}$ thymocytes do not survive CsA treatment and this subset contains the immediate precursors of SP thymocytes (Hugo et al., 1991b), it can be predicted that CsA should cause a relatively rapid loss of SP thymocytes, by preventing the generation of this subset. This explanation is feasible for CD4 SP thymocytes, because the subset shrank rapidly when CsA was administered, and lost continuity with the $CD4^+CD8^+CD3^{high}$ subset. The CD8 SPs, however, did not respond in this way for about 2 days, implying that it is the differentiation of their somewhat distant (rather than immediate) precursors that is inhibited by CsA. This implies that CD4 and CD8 SP thymocytes are not symmetrically derived from a common, CsA-sensitive precursor.

In BALB/c, there are two major subsets of $CD8^+CD3^+$ thymocytes that can be distinguished according to the expression of CD45RA. The $CD45RA^+$ subset, accounting for ~50% of the CD8 SPs, are essentially absent from some strains, such as C57BL/6 (Huby and Goff, 1992). They represent an additional subset when they are present (Goff and Huby, 1992), that is, there is no corresponding loss of $CD45RA^-$ cells. The $CD45RA^+$ (8RA) subset was found to respond to CsA with unusual kinetics; their numbers were unaffected for 10 days, after which they showed a rapid decline. This suggests that there is normally a significant flux of cells into the 8RA subset and that it is distantly derived from a CsA-sensitive precursor. The identity of this (and of their immediate) precursors remains enigmatic; the majority of DP thymocytes are too short-lived (Egerton et al., 1990) to be contenders, and a subset with such a rapid input of mature cells is unlikely to be of extrathymic origin. 8RAs may be derived from $CD8^+CD458RA^-$ cells, leading us to speculate that they may constitute thymic emigrants. However, intrathymic random cell labeling with FITC has revealed, as in strains such as CBA/Ca (Kelly and Scollay, 1990) that have low numbers of 8RAs (Huby and Goff, 1992), that the recent thymic emigrants of BALB/c are also $CD45RA^-$ (R. Scollay, personal communication). In B10.D2 mice, which essentially lack 8RAs, the CD8 SP subset responds to CsA with kinetics similar to those of the $CD45RA^-$ subset of CD8 SPs of the BALB/c (unpublished data).

CsA treatment can lead to the generation of autoreactive T cells capable of inducing a graft-versus-host like disease in immunocompromised animals (Cheney and Sprent 1983; Glazier et al., 1983; Hess et al., 1985). Autoreactive T cells can be found in the thymus of diseased animals (Beschorner et al., 1988b), where they are presumably generated during CsA treatment, because prior thymectomy is protective (Sorokin et al., 1986). The aetiology of the disease, however, may depend critically upon the destruction of thymic medullary stroma initiated by CsA-induced cytotoxic T cells (Beschorner et al., 1988b); T cells capable of recognizing self-superantigens (Kappler et al., 1987, 1988) have been found in the thymus (Gao et al., 1988; Jenkins et al., 1989) and periphery (Urdahl et al., 1992) of CsA-treated mice. This has led to the hypothesis that CsA treatment leads to a general failure of clonal deletion, allowing such cells to proceed beyond the immature $CD4^+CD8^+$ stage at which they are normally deleted (Hengartner et al., 1988; Hugo et al., 1991a), albeit in small numbers owing to CsA blocking the maturation of SP thymocytes.

Previously, it has been shown that CsA generally inhibits the ability of immature thymocytes to undergo apoptosis (Shi et al., 1989), the major mechanism whereby clonal deletion is achieved (Jenkinson et al., 1989). Using a flow cytometric method (Swat et al., 1991a, 1991b), we have demonstrated that CsA specifically affects the development of the apoptotic $CD4^{dim}CD8^{dim}$ phenotype amongst potentially autoreactive TCR-$V\beta3^+$ thymocytes in *Mtv-6*$^+$ mice. The loss of CD8, concomitant with up-regulation of TCR, was more greatly inhibited than that of CD4, and because apoptosis occurs rapidly, this is likely to reflect active regulation. Interestingly, it has been found that CsA fails to block the early phase of CD4 down-regulation induced by protein kinase C-activation, but effectively blocks CD8 down-regulation (Nakayama and Nakauchi, 1993). Nevertheless, acquisition of a $CD4^{dim}CD8^{dim}$ phenotype and population deletion did ultimately occur for immature TCR-$V\beta3^+$ thymocytes. Essentially, no mature TCR-$V\beta3^+$ thymocytes developed, as determined by three-color flow cytometry, a method capable of detecting them with greater accuracy and sensitivity than was possible in previous studies (Gao et al., 1988; Jenkins et al., 1989; Urdahl et al., 1992). This suggests that CsA effectively prevented the maturation of potentially autoreactive thymocytes that it had permitted to escape clonal deletion. TCR-Vβ3 deleted by *Mtv-6*, however, may be unusual in this respect; it is possible that the deletion of cells reactive to conventional self-antigens may be more effectively inhibited by CsA, allowing their maturation into autoreactive T cells.

To summarize, we find that CsA directly inhibits the development of $CD4^+Cd8^+CD3^{high}$ thymocytes. CD4 and CD8 SP thymocytes are derived by nonidentical pathways from these CsA-sensitive precursors. The $CD45RA^+$ subset of CD8 SP thymocytes, present in only some mouse strains, has a rapid turnover and is derived in about 10 days from a CsA-sensitive precursor, possibly via the $CD45RA^-$ subset of CD8 SPs. CsA treatment does not allow the development of TCR-$V\beta3^+$ thymocytes, despite the fact that their apoptosis induced by *Mtv-6* appears to be inhibited. It is now known that CsA can affect thymocyte function by binding to cyclophilin, a modulator of calcineurin activity (Fischer et al., 1989; Takahashi et al., 1989; Liu et al., 1991). It remains to be determined why CsA

blocks thymocyte differentiation at such specific points of development, although linkage to a requirement for TCR stimulation is emerging as a possible common theme.

### ACKNOWLEDGMENTS

We would like to thank Dr. Roland Scollay, Walter and Eliza Hall Institute, Melbourne, Australia, for supplying unpublished data from intrathymic labeling experiments and Professor Peter Beverley for his constructive criticism of the manuscript. This work was funded by the Imperial Cancer Research Fund, 44 Lincoln's Inn Fields, London, UK.

*(Received June 23, 1994)*

*(Accepted October 24, 1994)*

### REFERENCES

Abe R., Vacchio M.S., Fox B., and Hodes R.J. (1988). Preferential expression of the T-cell receptor V$\beta$ 3 gene by Mls$^c$ reactive T cells. Nature **335:** 827–830.

Beschorner W., Hess A.D., Schinn C., and Santos G. (1988a). Transfer of CsA-associated syngeneic GVHD by thymocytes. Resemblance to chronic GVHD. Transplantation **45:** 209.

Beschorner W.E., Olson J.L., Hess A.D., DiGennaro K.A., and Santos G.W. (1988b). Cyclosporine-induced cell-mediated injury of the thymic medullary epithelium. Transplantation **45:** 797.

Bryson J.S., Jennings C.D., Caywood B.E., and Kaplan A.M. (1989). Induction of a syngeneic graft-versus-host disease in DBA/2 mice. Transplantation **48:** 1042.

Cheney R.T., and Sprent J. (1983). Capacity of cyclosporine A to induce auto GVHD and impair intrathymic T cell differentiation. Transplant. Proc **17:** 528.

Choi Y., Marrack P., and Kappler J.W. (1992). Structural analysis of a mouse mammary tumour virus superantigen. J. Exp. Med. **175:** 847–852.

Clipstone N.A., and Crabtree G.R. (1992). Identification of calcineurin as a key signalling enzyme in T-lymphocyte activation. Nature **357:** 695–697.

Crispe I.N., Moore M.W., Husmann L.A., Smith L., Bevan M.J., and Shimonkevitz R.P. (1987). Differentiation potential of subsets of CD4-8- thymocytes. Nature **329:** 336–339.

Dellabona P., Peccoud J., Kappler J., Marrack P., Benoist C., and Mathis D. (1990). Superantigens interact with MHC class II molecules outside of the antigen groove. Cell **62:** 1115–1121.

Egerton M., Shortman K., and Scollay R. (1990). The kinetics of immature murine thymocyte development in vivo. Int. Immunol. **2:** 501–507.

Fischer G., Whittmann-Liebold B., Lang K., Kiefhaber T., and Schmid F.X. (1989). Cyclophilin and peptidyl-prolyl isomerase are probably identical proteins. Nature **337:** 476–478.

Frey J.R., Ernst B., Surh C.D., and Sprent J. (1992). Thymus-grafted scid mice show transient thymopoiesis and limited depletion of v-beta-11 + t-cells. J. Exp. Med. **175:** 1067–1071.

Fry A.M., Jones L.A., Kruisbeek A.M., and Mathis L.A. (1989). Thymic requirement for clonal deletion during T cell development. Science **246:** 1044–1046.

Gao E.K., Lo D., Cheney R., Kanagawa O., and Sprent J. (1988). Abnormal differentiation of thymocytes in mice treated with cyclosporin A. Nature **336:** 176–179.

Glazier A., Tutchka P.J., Farmer E.R., and Santos G.W. (1983). Graft-versus-host disease in cyclosporin A-treated mice after syngeneic and autologous reconstitution. J. Exp. Med. **158:** 1.

Goff L.K., and Hudy R.D.J. (1992). Characterisation of constitutive and strain-dependent subsets of CD45RA$^+$ cells in the thymus. Int. Immunol. **4:** 1303.

Hengartner, H., Odermatt B., Schneider R., Schreyer M., Walle G., MacDonald H.R., and Zinlermagel; R.M. (1988). Deletion of self-reactive T cells before entry into the thymus medulla. Nature **336:** 388–390.

Hess A.D., Horowitz L., Beschorner W.E., and Santos G.W. (1985). Development of graft-versus host disease-like syndrome in cyclosporin treated rats after syngeneic bone marrow transplantation: I. Development of cytotoxic T lymphocytes with apparent polyclonal anti-Ia specificity including autoreactivity. J. Exp. Med. **161:** 718.

Hodes R.J., Sharrow S.O., and Solomon A. (1989). Failure of T cell receptor $\beta$negative selection in an athymic environment. Science **246:** 1041–1044.

Huby R.D.J. (1990). Studies on the influence of Cyclosporine A on the development of thymocytes. Ph.D. thesis, University of London.

Huby R.D.J., and Goff L.K. (1992). The variable occurrence of CD45RA on CD8 + thymocytes correlates with the presence of *Mtv* sequences; its expression on other thymocytes is rare. Eur. J. Immunol. **22:** 1659–1662.

Huby R.D.J., Janossy G., and Lampert I.A. (1989). Cyclosporin A prevents the *in vivo* development of murine prothymocytes from uncommitted (Thy-1$^-$) precursor cells. Immunology **68:** 564–569.

Huesmann M., Scott B., Kisielow P., and von Boehmer H. (1991). Kinetics and efficacy of positive selection in the thymus of normal and T cell receptor transgenic mice. Cell **66:** 533–540.

Hugo P., Boyd R.L., Waanders G.A., Petrie H.T., and Scollay R. (1991a). Timing of deletion of autoreactive V$\beta$ 6$^+$ cells and down modulation of either CD4 or CD8 on phenotypically distinct CD4$^+$8$^+$subsets of thymocytes expressing intermediate or high levels of T cell receptor. Int. Immunol. **3:** 265–272.

Hugo P., Boyd R.L., Waanders G.A., and Scollay R. (1991b). CD4$^+$CD8$^+$CD3$^{high}$ thymocytes appear transiently during ontogeny: Evidence from phenotypic and functional studies. Eur. J. Immunol. **21:** 2655–2660.

Jenkins M.K., Schwartz R.H., and Pardoll D.M. (1989). Effects of Cyclosporine A on T cell development and clonal deletion. Science **241:** 1665.

Jenkinson E.J., Kingston R., Smith C.A., Williams G.T., and Owen J.J. (1989). Antigen-induced apoptosis in developing T cells: A mechanism for negative selection of the T cell receptor repertoire. Eur. J. Immunol. **19:** 2175–2177.

Kai O., and Franklin R.M. (1983). Effects of cyclosporine A on mouse lymphoid tissues. Brit. J. Exp. Path. **64:** 534.

Kanariou M., Huby R.D.J., Ladyman H.M., Colic M., Silovapenko G., Lampert I.A., and Ritter M.A. (1989). Structure of the thymic epithelial cell microenvironment before and after immunosuppression with cyclosporine A. *Clin. Exp. Immunol.* **78:** 263.

Kappler J.W., Roehm N., and Marrack P. (1987a). T cell tolerance by clonal elimination in the thymus. Cell **49:** 273–280.

Kappler J.W., Staerz U., White J., and Marrack P.C. (1988). Self-tolerance eliminates T cells specific for Mls-modified products of the major histocompatibility complex. Nature **332:** 35–40.

Kappler J.W., Wade T., White J., Kushnir E., Blackman M., Bill J., Roehm N., and Marrack P. (1987b). A T cell receptor V beta segment that imparts reactivity to a class II major histocompatibility complex product. Cell **49:** 263–271.

Kelly K.A., and Scollay R. (1990). Analysis of recent thymic emigrants with subset- and maturity-related markers. Int. Immunol. **2:** 419–425.

Liu J., Farmer J.D., Lane W.S., Friedman J., Weissman I., and Schreiber S.L. (1991). Calcineurin is a common target of cyclophilin-cyclosporin A and FKBP-FK506 complexes. Cell **66:** 807.

MacDonald H.R., Schneider R., Lees R.K., Howe R.C., Acha O.H., Festenstein H., Zinkernage R.M., and Hengartner H. (1988). T-cell receptor V beta use predicts reactivity and tolerance to Mlsa-encoded antigens. Nature **332:** 40–45.

Nakayama K.-I., and Nakauchi H. (1993). Cyclosporin A inhibits the decrease of CD4/CD8 expression induced by protein kinase C activation. Int. Immunol. **5:** 419–426.

O'Keefe S.J., Tamura J., Kincaid R.L., Tocci M.J., and O'Neill E.A. (1992). FK-506-and CsA-sensitive activation of the interleukin-2 promotor by calcineurin. Nature **357:** 692–694.

Penit C. (1990). Positive selection is an early event in thymocyte differentiation: High TCR expression by cycling immature thymocytes precedes final maturation by several days. Int. Immunol. **2:** 629–638.

Pullen A.M., Marrack P., and Kappler J.W. (1988). The T-cell repertoire is heavily influenced by tolerance to polymorphic self-antigens. Nature **335:** 796–801.

Sakaguchi S., and Sakaguchi N. (1988). Thymus and autoimmunity. Transplantation of the thymus from cyclosporin A-treated mice causes organ-specific autoimmune disease in athymic nude mice. J. Exp. Med. **167:** 1479–1485.

Sakaguchi S., and Sakaguchi N. (1989). Organ-specific autoimmune disease induced in mice by elimination of T cell subsets. V. Neonatal administration of cyclosporin A causes autoimmune disease. J. Immunol. **142:** 471–480.

Schwartz R.H. (1989). Acquisition of immunologic self-tolerance. Cell **57:** 1073–1081.

Scollay R., Wilson A., D'Amico A., Kelly K., Egerton M., Pearse M., Wu L., and Shortman K. (1988). Developmental status and reconstitution potential of subpopulations of murine thymocytes. Immunol. Rev. **104:** 81–120.

Sha W.C., Nelson C.A., Newberry R.D., Kranz D.M., Russell J.H., and Loh D.Y. (1988). Positive and negative selection of an antigen receptor on T cells in transgenic mice. Nature **336:** 73–76.

Shi Y.F., Sahai B.M., and Green D.R. (1989). Cyclosporin a inhibits activation-induced cell death in T-cell hybridomas and thymocytes. Nature **339:** 625–626.

Shortman K., Wilson A., Egerton M., Pearse M., and Scollay R. (1988). Immature CD4− CD8+ murine thymocytes. Cell Immunol. **113:** 462–479.

Sorokin R., Kimura H., Schroder K., Wilson D.H., and Wilson D.B. (1986). Cyclosporine-induced immunity. Conditions for expressing the disease, requirement for intact thymus and potency estimates of autoreactive lymphocytes in drug-treated rats. J. Exp. Med. **164:** 1615.

Swat W., Ignatowicz L., and Kisielow P. (1991a). Detection of apoptosis of immature $CD4^{+}8^{+}$ thymocytes by flow cytometry. J. Immunol. Meth. **137:** 79–87.

Swat W., Ignatowicz L., von Boehmer H., and Kisielow P. (1991b). Clonal deletion of immature $CD4^{+}8^{+}$ thymocytes in suspension culture by extrathymic antigen-presenting cells. Nature **351:** 150–153.

Takahama Y., Kosugi A., and Singer A. (1991). Phenotype, ontogeny, and repertoire of $CD4^{-}CD8^{-}$ T cell receptor alpha beta + thymocytes. Variable influence of self-antigens on T cell receptor V beta usage. J. Immunol. **146:** 1134–1141.

Takahashi N., Hayano T., and Suzuki M. (1989). Peptidyl-prolyl cis-trans isomerase is the cyclosporin A-binding protein cyclophilin. Nature **337:** 473–475.

Urdahl K.B., Pardoll D.M., and Jenkins M.K. (1992). Self-reactive T cells are present in the peripheral lymphoid tissues of cyclosporin A-treated mice. Int. Immunol. **4:** 1341–1349.

Wodzig K.W., Majoor G.D., and van Breda Vriesman P.J. (1991). On the localization of effector cells in cyclosporin-induced autoimmunity. Autoimmunity **10:** 275–283.

Wyllie A.H., Morris R.G., Smith A.L., and Dunlop D. (1984). Chromatin cleavage in apoptosis: Association with condensed chromatin morphology and dependence on macromolecular synthesis. J. Pathol. **142:** 67–77.

